# Development of a Real-Time PCR Assay for the Detection of Donkey (*Equus asinus*) Meat in Meat Mixtures Treated under Different Processing Conditions

**DOI:** 10.3390/foods9020130

**Published:** 2020-01-26

**Authors:** Mi-Ju Kim, Seung-Man Suh, Sung-Yeon Kim, Pei Qin, Hong-Rae Kim, Hae-Yeong Kim

**Affiliations:** Department of Food Science and Biotechnology, Institute of Life Sciences & Resources, Kyung Hee University, Yongin 17104, Korea; mijukim79@gmail.com (M.-J.K.); teri2gogo@naver.com (S.-M.S.); sungyeon94@naver.com (S.-Y.K.); qinp77@163.com (P.Q.); star6783204@nate.com (H.-R.K.)

**Keywords:** food adulteration, food fraud, donkey, *cytochrome b*, real-time PCR, meat products

## Abstract

In this study, a donkey-specific primer pair and probe were designed from mitochondrial *cytochrome b* gene for the detection of raw donkey meat and different processed meat mixtures. The PCR product size for donkey DNA was 99 bp, and primer specificity was verified using 20 animal species. The limit of detection (LOD) was examined by serially diluting donkey DNA. Using real-time PCR, 0.001 ng of donkey DNA could be detected. In addition, binary meat mixtures with various percentages of donkey meat (0.001%, 0.01%, 0.1%, 1%, 10%, and 100%) in beef were analyzed to determine the sensitivity of this real-time PCR assay. At least 0.001% of donkey meat was detected in raw, boiled, roasted, dried, grinded, fried, and autoclaved meat mixtures. The developed real-time PCR method showed sufficient specificity and sensitivity in identification of donkey meat and could be a useful tool for the identification of donkey meat in processed products.

## 1. Introduction

Identification of animal species in meat products is important for preventing food adulteration and providing accurate information regarding meat species to consumers. Donkey meat products are highly nutritious; moreover, in many countries, including Korea, it is considerably more expensive than other meats owing to its low supply [1]. In Islamic countries, donkey meat consumption is prohibited on religious grounds [2]. Due to donkey meat being expensive, it is likely to be mixed with other cheaper meats for economic benefits, and there is a need to avoid donkey meat entering the food chain in Islamic countries. Therefore, it is necessary to develop reliable and specific detection methods that can accurately identify animal species from meat products to prevent cases of disguising meat from one species as another [3,4]. 

To date, many protein- and DNA-based detection methods have been developed to determine animal species in food products. In particular, DNA-based methods have been used to detect target species in processed foods because DNA is stable at high temperatures and pressures [5,6,7]. Real-time PCR is an effective tool that accurately amplifies target DNA. Several real-time PCR methods, particularly based on detection via TaqMan probes, have been developed with high sensitivity and accuracy to distinguish common meat species such as pork, lamb, and beef [8,9,10,11,12].

Mitochondrial DNA (mtDNA) has been mainly used to detect target species in meat products, and mtDNA sequences from related species have been phylogenetically studied [13]. In addition, because mtDNA evolves faster than nuclear DNA, mtDNA has been used to discriminate target species from similar species. Further, mitochondria are present in high copy numbers in cells. Thus, real-time PCR based on specific mtDNA sequences can amplify target DNA degraded by food processing or mixed with other species [14,15]. In many studies, mitochondrial *cytochrome b* has been used to develop species-specific real-time PCR detection methods [12,16,17].

Here, we designed a donkey-specific primer and probe from mitochondrial *cytochrome b* and developed a real-time PCR method to accurately identify donkey meat. Although there have been several previous studies for donkey meat detection [1,9], no study has applied donkey meats treated under a variety of processing conditions. Thus, in this study, we evaluated the applicability of the developed method for the detection of donkey meat using raw, boiled, roasted, dried, grinded, fried, and autoclaved meat mixtures.

## 2. Materials and Methods

### 2.1. Preparation of Samples and Binary Meat Mixtures

A total of 20 raw meat samples were obtained from the Conservation Genome Resource Bank for Korean Wildlife (CGRB), the National Institute of Animal Science (NIAS), and local markets in South Korea. All samples were homogenized in small pieces and stored at −20 °C until analysis. 

Binary meat mixtures were prepared to determine the detection limit of donkey-specific real-time PCR assay. For binary raw meat mixtures, 10 g of each of donkey and beef was lyophilized for 24 h using a freeze dryer (Ilsin Biobase, Dongduchon, Korea) to remove moisture of raw meats without DNA degradation, and then ground. In addition, to evaluate the applicability of the developed method in processed meat products, two meats were treated under six different processing conditions as follows: 1) boiled at 100 °C for 15 min in water bath (MONO-TECH, Daegu, Korea), 2) roasted at 200 °C for 5 min in hot plate (Corning Co., New York, NY, USA), 3) dried at 65 °C for 12 h in dry oven (HANKUK S&I, Hwaseong, Korea), 4) grinded for 5 min in commercial grinder (Buwon Electronics, Daegu, Korea), 5) fried at 180 °C for 5 min in cooking oil, and 6) autoclaved at 121 °C 150 kPa for 30 min. Each meat for the six different mixtures was prepared in triplicate, which was made on different days and from meats of different origins. After treatments, each of binary meat mixtures containing six different percentages (0.001%, 0.01%, 0.1%, 1%, 10%, and 100% (*w*/*w*)) of donkey meats in beef was prepared. Samples (final weight, 100 mg) of various meat mixtures were used for analysis.

### 2.2. DNA Extraction

Genomic DNA was extracted from raw and autoclaved meat mixtures using the DNeasy Blood and Tissue kit (Qiagen, Hilden, Germany), following the manufacturer’s instructions with minor modifications. Briefly, 100 mg of each sample was lysed with 3600 µL of ATL buffer and 400 µL of proteinase K (20 mg/mL) in a water bath at 56 °C for 1 h. After adding 40 µL of RNase A (100 mg/mL), the mixture was incubated at room temperature for 2 min. AL buffer (4000 µL) and 100% ethanol (4000 µL) were mixed with the DNA mixture, and the sample was centrifuged through a spin column. After washing with AW1 and AW2 buffers, the column-bound DNA was eluted with purified water. The purity and concentration of the extracted DNA were confirmed using a Maestro Nano spectrophotometer (Maestrogen, Las Vegas, NV, USA).

### 2.3. Primer and Probe Design

A donkey-specific primer pair and probe for the detection of donkey were designed to amplify the specific target DNA (Table 1). To design a donkey-specific primer pair, mitochondrial *cytochrome b* sequences from 20 animal species, including donkey, beef, and horse (Accession No.: FJ428510.1, D34635.1, and MH594485.1, respectively) were obtained from GenBank. All sequences were aligned using Clustal Omega program (http://www.ebi.ac.uk/Tools/msa/clustalo/). The primer pair and probe were designed using the Primer Designer program version 3.0 (Scientific and Education Software, Durham, NC, USA) and synthesized by Bionics (Seoul, Korea) and Bioneer (Daejeon, Korea).

### 2.4. Conventional PCR Reaction

Conventional PCR was performed using a thermal cycler (PC808, ASTEC, Kyoto, Japan) under the following conditions: pre-incubation at 94 °C for 5 min, 30 cycles of denaturation for 30 s at 94 °C, annealing for 30 s at 60 °C, extension for 30 s at 72 °C, and final extension for 5 min at 72 °C. The PCR reaction mixture comprised 400 nM of each primer, 0.5 U of Ampli-Gold Taq polymerase (Applied Biosystems, Foster City, CA, USA), 10× PCR buffer (Applied Biosystems), 2.5 mM of each dNTP (Applied Biosystems), 1.5 mM of MgCl_2_ (Applied Biosystems), and 10 ng of DNA template isolated from each of animal species for the specificity test in a total reaction volume of 25 µL. All PCR products were electrophoresed on a 2% agarose gel and then visualized under UV irradiation.

### 2.5. Real-Time PCR Reaction

Real-time PCR amplification was performed using an ABI 7500 Real-time PCR instrument (Applied Biosystems). The PCR reaction was performed in a final volume of 25 µL, containing 2× TaqMan Universal Master mix (Applied Biosystems), 400 nM of primer pairs, 200 nM of the probe, and 10 ng of the DNA template. Real-time PCR was performed with a holding stage at 95 °C for 10 min, followed by 40 cycles at 95 °C for 15 s and 60 °C for 1 min. All real-time PCR reactions were performed in triplicates; no-template control (NTC) was used as a negative control. Data were analyzed using 7500 Software V.2.3 (Applied Biosystems).

### 2.6. Specificity and Sensitivity of Real-Time PCR

The specificity of the donkey-specific primer pair and probe was tested using 10 ng of genomic DNA extracted from 20 animal species. To confirm the presence of DNA, endogenous primer pair and probe targeting the 18S rRNA gene were also used [18]. The sensitivity of the real-time PCR was measured using 10-fold serially diluted DNA (from 10 to 0.001 ng) extracted from donkey. The detection limit of real-time PCR in 6 processed binary mixtures containing donkey meat (ranging in concentration from 10% to 0.001%) mixed with beef meat was used.

## 3. Results and Discussions

### 3.1. Specificity

The donkey-specific primer and probe were designed to get a small product size of 99 bp from mitochondrial *cytochrome b*. The specificity of donkey primer set was confirmed using the DNA from 20 animal species as templates for this assay. Only the DNA fragment specific for donkey was amplified by conventional PCR, and there was no amplification in 19 nontarget species (Table 2). The PCR product amplified by the donkey-specific primer was sequenced to verify the donkey species (Figure S1). The specificity of the real-time PCR method was additionally verified, and the donkey DNA was specifically amplified without any cross-reactivity against the 19 other animal species tested (Table 2). To confirm the presence of DNA, eukaryotic PCR targeting the 18S rRNA gene was performed. As shown in Table 2, positive signals were observed in all PCR reactions. Thus, our results proved that the donkey-specific primer and probe were accurately amplified the target DNA.

### 3.2. Sensitivity of the Donkey-Specific Real-Time PCR Assay

**Figure 1:**
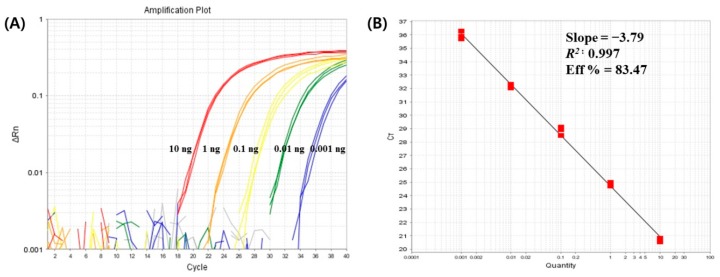
Amplification plot (**A**) and standard curve (**B**) for the detection of donkey DNA using 10-fold serial dilutions (from 10 to 0.001 ng).

The sensitivity of the donkey-specific real-time PCR targeting *cytochrome b* gene was determined using 10-fold serially diluted donkey DNA from 10 to 0.001 ng. Ct values were plotted against logarithmic DNA concentrations to construct the standard curve for the donkey DNA. The slope and correlation coefficient (R^2^) of the standard curve were −3.79 and 0.997, respectively. PCR efficiency was calculated using the equation “E = (10^(−1/slope)^ − 1)” and was determined to be 83.47% (Figure 1). Each PCR reaction was performed thrice, and 0.001 ng of the donkey DNA was detected in all the reactions. The absolute detection limit of the donkey-specific real-time PCR assay was as low as 0.001 ng. These results demonstrated that the real-time PCR method developed in this study has good linearity and sensitivity. 

### 3.3. Application of the Real-Time PCR Assay to Meat Mixtures Treated under Different Processing Conditions

The meat mixtures treated under six conditions were used to confirm the applicability of the developed method using highly processed meat products as well as raw meat, and various concentrations of binary meat mixtures were prepared for the limit of detection (LOD) test of this method. As shown in Table 3, 0.001% of donkey meat was successfully detected in all processed meat mixtures, despite high heat and pressure treatments of donkey meat. The average Ct values of three replicates using donkey DNA were 18.45 ± 0.7, 20.24 ± 0.97, 18.74 ± 0.06, 18.59 ± 0.31, 19.17 ± 0.6, 21.17 ± 0.55, and 20.86 ± 0.26 for raw, boiled, roasted, dried, ground, fried, and autoclaved meats, respectively. Ct values of the target species in the boiled, fried, and autoclaved meat mixtures were relatively higher than in other meat mixtures; this may be attributable to the fact that the DNA was degraded under the high pressure and temperature treatments [7,19]. 

The lowest percentage of donkey meat adulteration that could be detected by the real-time PCR method developed in this study was 0.001%, which was lower than 1% of detection limit reported by Chen et al. [1] and same or lower than 0.001% and 0.01% of detection limits reported by Kesmen et al. [19]. Therefore, this real-time PCR method can help to confirm the presence of donkey meat in highly processed meat products and provide accurate information on target meat species. For a more efficient detection method tool, a further study can be performed the development of multiplex real-time PCR for the detection of two genes, including the endogenous 18S rRNA gene. 

## 4. Conclusions

This study described the development of a real-time PCR method to identify donkey DNA. By targeting a 99 bp fragment of mitochondrial *cytochrome b*, the designed primer pair and probe specifically amplified the donkey DNA. The standard curve of the developed real-time PCR method has good linearity and sensitivity, which is adequate to successfully amplify the target DNA. Raw and highly processed meat mixtures were analyzed with a sensitivity of 0.001% to demonstrate the applicability of the method developed in the present study for detecting donkey meat in processed meat products. The applicability of this method was verified with six processing conditions that can be used for meat processing, and the applicability was confirmed under all processing conditions. Therefore, the real-time PCR method developed in this study could be a useful tool for the detection of donkey and determination of intentional adulterations or food fraud in highly processed meat products.

## Figures and Tables

**Table 1:** Sequences of primers and probes used in this study.

Primer Name	Sequences (5′→3′)	Target Genes	Amplicon Size (bp)	Reference
Don3 F	CGCTCCATTCCCAACAAACTAGGTGGT	*Cytochrome b*	99	This study
Don3 R	GCTTCGTTGTTTTGACATGTGTAGGGTA			
Don3 P	FAM-GCCCTTATCCTTTCCATCTTAATCC-TAMRA			
18SpEU-DIR	GGTAGTGACGAAAAATAACAATACAGGAC	18S rRNA	141	[18]
18SpEU-INV	ATACGCTATTGGAGCTGGAATTACC			
18S probe	FAM-AAGTGGACTCATTCCAATTACAGGGCCT-TAMRA			

**Table 2:** Specificity results using conventional and real-time PCR assays.

Common Name	Scientific Name	Conventional PCR		Real-Time PCR	
		Donkey-Specific PCR	Eukaryotic PCR	Donkey-Specific PCR	Eukaryotic PCR
Donkey	*Equus asinus*	+	+	+	+
Horse	*Equus caballus*	−	+	−	+
Beef	*Bos taurus*	−	+	−	+
Lamb	*Ovis aries*	−	+	−	+
Goat	*Capra hircus*	−	+	−	+
Deer	*Cervus elaphus*	−	+	−	+
Pork	*Sus scrofa domestica*	−	+	−	+
Rabbit	*Oryctolagus cuniculus*	−	+	−	+
Raccoon dog	*Nyctereutes procyonoides*	−	+	−	+
Dog	*Canis lupus familiaris*	−	+	−	+
Cat	*Felis catus*	−	+	−	+
Siberian chipmunk	*Tamias sibiricus*	−	+	−	+
Turkey	*Meleagris gallopavo*	−	+	−	+
Ostrich	*Struthio camelus*	−	+	−	+
Chicken	*Gallus gallus*	−	+	−	+
Pheasant	*Phasianus colchicus*	−	+	−	+
Duck	*Anas platyrhynchos*	−	+	−	+
Goose	*Anser anser*	−	+	−	+
Pigeon	*Columba livia domestica*	−	+	−	+
Japanese quail	*Coturnix japonica*	−	+	−	+

**Table 3:** Results of the developed real time-PCR method when applied to six meat mixtures processed following different technologies.

Target Species	Ratio of Donkey Meat in the Binary Meat Mixture (%)	Ct Values						
		Raw	Boiled	Roasted	Dried	Grinded	Fried	Autoclaved
**Donkey**	100	18.45 ± 0.70 ^a^	20.24 ± 0.97	18.74 ± 0.06	18.59 ± 0.31	19.17 ± 0.60	21.17 ± 0.55	20.86 ± 0.26
	10	21.55 ± 0.52	23.34 ± 0.64	21.79 ± 0.08	21.27 ± 0.25	22.30 ± 0.57	24.01 ± 0.33	23.86 ± 0.21
	1	24.58 ± 0.47	26.4 ± 0.63	24.97 ± 0.0.8	24.35 ± 0.43	25.71 ± 0.10	27.31 ± 0.42	26.68 ± 0.30
	0.1	27.37 ± 1.03	29.15 ± 1.06	28.19 ± 0.07	27.47 ± 0.43	28.84 ± 0.10	30.61 ± 0.38	29.53 ± 0.19
	0.01	30.48 ± 1.03	32.11 ± 1.20	31.32 ± 0.05	30.63 ± 0.56	31.83 ± 0.43	33.71 ± 0.27	32.67 ± 0.08
	0.001	33.59 ± 1.08	34.89 ± 0.96	34.35 ± 0.07	33.57 ± 0.51	34.13 ± 0.51	36.72 ± 0.33	35.69 ± 0.17

^a^ Average Ct value ± standard deviation obtained from triplicate reactions.

## Supplementary Materials

The following are available online at https://www.mdpi.com/2304-8158/9/2/130/s1, Figure S1: The identity result of sequences of the PCR products for donkey-specific primer sets.
