# Supplementary material for: Development of a Real-Time PCR Assay for the Detection of Donkey (Equus asinus) Meat in Meat Mixtures Treated under Different Processing Conditions
Source: Foods. 2020 Jan 26;9(2):130. doi: 10.3390/foods9020130 (PMC7074451; doi:10.3390/foods9020130)
Supplement: Supplementary file 1 [file foods-09-00130-s001.pdf]

Supplementary Data

Equus asinus isolate 3 breed Yunnan donkey cytochrome b (cytb) gene, partial cds; mitochondrial

Sequence ID: KT829585.1 Length: 1137 Number of Matches: 1

Range 1: 844 to 942 GenBank Graphics ▼ Next Match ▲ Previous Match

Score	Expect	Identities	Gaps	Strand
183 bits(99)	4e-43	99/99(100%)	0/99(0%)	Plus/Plus

```
Query  1    CGCTCCATTCCCAACAAACTAGGTGGTGTATTAGCCCTTATCCTTTCCATCTTAATCCTA  60
            ||||||||||||||||||||||||||||||||||||||||||||||||||||||||||||
Sbjct  844  CGCTCCATTCCCAACAAACTAGGTGGTGTATTAGCCCTTATCCTTTCCATCTTAATCCTA  903

Query  61   GCACTCATCCCTACCCTACACATGTCAAAACAACGAAGC  99
            |||||||||||||||||||||||||||||||||||||||
Sbjct  904  GCACTCATCCCTACCCTACACATGTCAAAACAACGAAGC  942
```

Figure S1. The identity result of sequences of the PCR products for donkey-specific primer sets.
